# 3D Printed Calcium Phosphate Cement (CPC) Scaffolds for Anti-Cancer Drug Delivery

**DOI:** 10.3390/pharmaceutics12111077

**Published:** 2020-11-11

**Authors:** Yan Wu, Lisa Woodbine, Antony M. Carr, Amit R. Pillai, Ali Nokhodchi, Mohammed Maniruzzaman

**Affiliations:** 1 Pharmaceutics Research Laboratory, Arundel Building, School of Life Sciences, University of Sussex, Falmer, Brighton BN1 9QJ, UK; yw354@sussex.ac.uk; 2 Sussex Centre for Genome Damage and Stability (GDSC), School of Life Sciences, University of Sussex, Falmer, Brighton BN1 9RQ, UK; l.j.woodbine@sussex.ac.uk (L.W.); a.m.carr@sussex.ac.uk (A.M.C.); 3 Pharmaceutical Engineering and 3D Printing (PharmE3D) Lab, Division of Molecular Pharmaceutics and Drug Delivery, College of Pharmacy, University of Texas at Austin, Austin, TX 78712, USA; arpillai@utexas.edu

**Keywords:** 3D bio-printing, tissue engineering, anti-cancer, scaffolds, dissolution, 5-FU, cancer cell culture

## Abstract

One of the main applications of bone graft materials is filling the gap after the surgical removal of bone cancer or tumors. Insufficient healing commonly leads to non-union fracture which could lead to cancer resurgence or infection. Emerging 3D printing of on-demand bone graft biomaterials can deliver personalized solutions with minimized risk of relapse and recurrence of cancer after bone removal surgery. This research aims to explore 3D printed calcium phosphate cement (CPC) based scaffolds as novel anti-cancer drug delivery systems to treat bone cancer. For the study, various 3D printed CPC based scaffolds (diameter 5 mm) with interconnected pores were utilized. Various optimized polymeric solutions containing a model anticancer drug 5-fluorouracil (5-FU) was used to homogenously coat the CPC scaffolds. Both hydrophilic Soluplus (SOL) and polyethylene glycol (PEG) and a combination of both were used to develop stable coating solutions. The surface morphology of the coated scaffolds, observed via SEM, revealed deposition of the polymeric solution represented by a semi-smooth surface as opposed to the blank scaffolds that showed a smoother surface. An advanced surface analysis conducted via confocal microscopy showed a homogenous distribution of the drug throughout the coated scaffolds. Solid-state analysis studied by applying differential scanning calorimetry (DSC) and X-ray diffraction (XRD) revealed semi-crystalline nature of the drug whereas mechanical analysis conducted via texture analysis showed no evidence in the change of the mechanical properties of the scaffolds after polymeric solutions were applied. The FTIR analysis revealed no major intermolecular interactions between 5-FU and the polymers used for coatings except for F2 where a potential nominal interaction was evidenced corresponding to higher Soluplus content in the formulation. In vitro dissolution studies showed that almost 100% of the drug released within 2 h for all scaffolds. Moreover, in vitro cell culture using two different cell lines (Hek293T-human kidney immortalized cell line and HeLa-human bone osteosarcoma epithelial cell line) showed significant inhibition of cell growth as a function of decreased numbers of cells after 5 days. It can be claimed that the developed 5-FU coated 3D printed scaffolds can successfully be used as bone graft materials to potentially treat bone cancer or bone neoplasm and for personalized medical solutions in the form of scaffolds for regenerative medicine or tissue engineering applications.

## 1. Introduction

Three-dimensional bioprinting (3D bioprinting) is a subclass of additive manufacturing (AM) for printing bioactive 3D tissues and organs layer by layer using cell compatible or cell loaded bio-materials [1,2,3]. The fabrication of 3D constructs can be fast achieved in any computer-designed structure automatically in the 3D bioprinting process. Recently, there are many research achievements in the 3D bioprinting sphere such as the research published by Parka and his colleagues where they printed an organ-on-a-chip which can add complex stimuli in vitro tissue models to better recapitulate living human tissues [4]. Another key milestone in the realm of 3D bioprinting comes from Kang et al. who fabricated a muscle fiber-like bundle structures with poly(ε-caprolactone) (PCL) pillars applying a micro-extrusion bioprinting system [5]. Further expanding on the research conducted by Kang et al., 3D aligned-muscle constructs with PCL-geometrical constraints were also created by the same technique [6]. The synergistic application of a hybrid 3D cell printing system combined with extrusion and ink-jet modules were also used for making a 3D skin model composed of a fibroblast-populated 3D dermal layer using a transwell system as well as with keratinocytes of the epidermal layer [7]. There are several different 3D bioprinting techniques which can be categorized as ink-jet bioprinting systems (which is also known as droplet-based printing techniques), laser-assisted bioprinting systems and extrusion-based bioprinting systems [8,9]. Amongst those techniques, extrusion-based bioprinting system has been utilized for the printing of bone grafts because of cost-effectiveness as well as fast plotting.

Calcium phosphate cement (CPC) has appeared as an emerging bone-filling material to promote bone formation and growth which has been demonstrated in various orthopedic and dental applications (e.g., maxillary bone augmentations) [10]. CPCs have several advantages over other bone-filling materials such as setting at body temperature and being mostly injectable as well as malleable. CPCs are generally composed of one or several Ca-P phases (e.g., tetracalcium phosphate, dicalcium phosphate anhydrous) present in powder form. When CPC is mixed with an aqueous solution, it forms a solid structure (set cement) with superior biocompatibility and bioactivity compared to other synthetic bone-filling materials (e.g., polymers). There are various studies reported on the enhancement of the biological performance such as biocompatibility, osteoconductivity, osteoinductivity, biodegradability, and interactions with cells of CPCs [11]. A recent study authored by Kilian et al. 2020, investigated the development of a pasty CPC alongside another alginate-based bioink for the fabrication of 3D printed construct with high shape fidelity to reconstruct osteochondral tissue layers. The study concluded that the presence of a mineralized zone in the fabricated constructs potentially interfered with chondrogenesis and was found to support chondrogenic ECM production [12]. Similarly, Ahlfeld et al., 2020, investigated a two-fold study in which a novel plasma-based bioink was combined with a printable self-setting CPC to fabricate bone-like tissue constructs. The authors concluded that their developed novel bioink was a promising platform for tissue engineering applications supplemented with the combination of CPC for enhanced bioprinted bone-like constructs [13]. Trombetta et al. 2020, reported 3D printing of bioresorbable CPC scaffolds for sustained antimicrobial drug release and investigated its efficacy of femoral implant-associated osteomyelitis in vivo. The results indicated that 3D printed CPC scaffolds loaded with antimicrobial agents showed better bone growth in a single-stage modification as opposed to traditional two-stage modifications [14]. However, most of the reported studies have either focused on the use of CPC or materials optimization. But none or very few of them have investigated the potential use of 3D printed CPC scaffolds for anti-cancer drug delivery and tissue engineering for bone cancer treatment.

**Figure 1:**
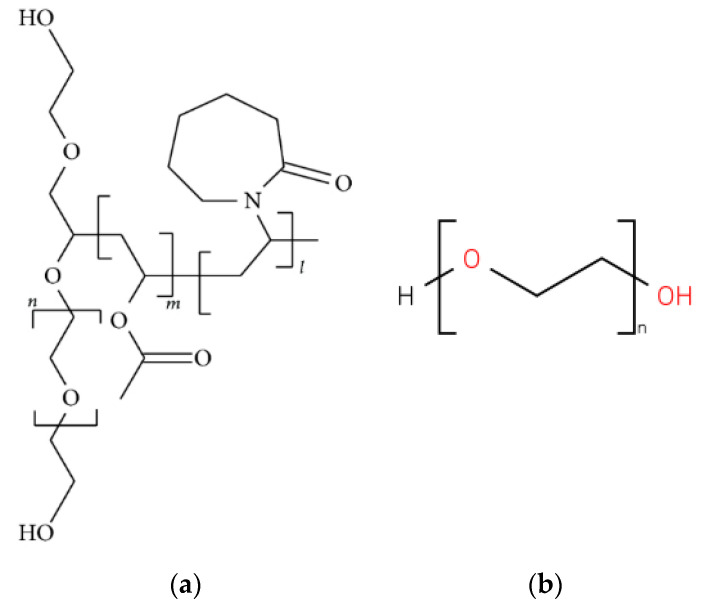
Chemical structure of the excipients (**a**) Soluplus^®^ and (**b**) polyethylene glycol (PEG).

5-fluorouracil (5-FU) a widely used anti-cancer drug (pKa = 8.02, logp = −0.89) with a half-life of 8 to 20 min [15] was chosen as a model drug in this experiment because of its popularity and low cost. Due to poor solubility of 5-FU in deionized water (less than 1 mg/mL at 19 °C [16]), Soluplus^®^ (polyvinyl caprolactam-polyvinyl acetate-polyethylene glycol graft copolymer (PCL-PVAc-PEG)) and polyethylene glycol (PEG) were added to 5-FU solutions as solubility enhancers and their chemical structures are shown in Figure 1. Soluplus^®^ (Figure 1a) is an innovative commercial excipient for improving the bioavailability and solubility of the active ingredients [17]. It has been widely used in the extrusion process because of its high flowability and excellent extrudability [18]. PEG (Figure 1b) is a polyether compound which is commonly utilized in the pharmaceutical industry, and it was chosen to be a good excipient for 5-FU formulations because of its hydrophilic nature.

One of the main applications of bone grafts is filling the gap in bone after surgical removal of bone cancer tumors. While it is common that patients with bone cancer face the risk of relapse and recurrence after bone removal surgery. It commonly occurs due to the presence of non-union fractures which is a result of the inefficiency of bone healing in certain scenarios. Especially due to the large gaps left after tumor resection [19]. Tumor recurrence after placement of bone filling biomaterials remains one of the major causes of biomaterials failure in dental and orthopedic applications [20]. A local release of an anti-cancer agent from a bone implant that can provide a controlled release of the agent in surrounding cancerous tissue could be an innovative area of research for preventing recurrence after placement of dental/orthopedic biomaterials. In situ delivery of an anti-cancer agent could potentially provide adequate therapeutic dosage while minimizing the side effects of the anti-cancer agent in the nearby uninfected tissues. Currently, there is no available bone graft material for clinical use with anti-cancer properties that can reduce the risk of bone cancer resurgence and/or prevent the spread of cancer to other organs. A bone graft material that possesses tunable anti-cancer properties has numerous advantages over the current graft materials used in the clinic [21].

This research aims to develop a novel anti-cancer drug-coated calcium phosphate cement (CPC) scaffold to potentially decrease the relapse and resurgence of bone cancer after surgery. To the best of our knowledge, there is no anti-cancer drug-coated CPC scaffold currently available for commercial use. There has been some previous work about drug-coated 3D printed scaffolds in other matrices, such as silk fibroin, alginate, Pluronic, polymeric matrices [22,23,24]. Owing to the superior advantages of CPCs such as rapidly setting at body temperature, we believe 3D printed CPC scaffold can be advantageous and more suitable material for the anti-cancer drug delivery systems. This can also lead to numerous potential applications of these emerging materials in drug delivery and tissue engineering applications. For this purpose, 3D bioprinted CPC scaffolds were coated with different 5-FU formulations to be utilized as an anti-cancer drug delivery system. The coating ingredients were tested by differential scanning calorimetry (DSC). Surface analysis and texture analysis were conducted for these coated scaffolds to investigate the mechanical properties of the scaffolds before and after coating. The efficiency of the scaffold was tested by investigating its ability to kill the cancer cells in vitro.

## 2. Materials and Methods

### 2.1. Materials

CPC scaffolds with interconnected pores (diameter 5mm, thickness 2 mm) were printed by a semi-solid extrusion 3D printer (Innotere, Radebeul, Germany) using calcium phosphate semi-solid paste (α-tricalcium phosphate and calcium-deficient hydroxyapatite). All 3D printed scaffolds were subjected to coating using a polymeric solution of a model anti-cancer drug 5-Fluorouracil (5-FU, Acros Organics™, 99%, Fair Lawn, NJ, USA). Soluplus^®^ (BASF Ltd., London, UK) and polyethylene glycol (PEG 6000, Acros Organics™, Ludwigshafen, Germany) were used. We used 0.9% NaCl solution as the dissolution medium in the dissolution test. Cancer and transformed cell lines, HeLa and HEK293T, stably expressing GFP constructs were generated at the GDSC, University of Sussex, Brighton, UK. Cell lines were maintained in DMEM supplemented with 10% FCS, penicillin/streptomycin and L-Glutamine at 37 °C and 5% CO_2_. PBS (pH = 7.4, Fisher Scientific, Loughborough, UK) was used for washing.

### 2.2. Methods

#### 2.2.1. The Preparation of 5-FU Coating Formulations

Formulations containing 5-FU were prepared by adding 5-FU powder into DI water under stirring at 80 °C until it was fully dissolved. The ingredients of formulations F1 to F3 are illustrated in Table 1. Soluplus^®^ and PEG 6000 were added to 5-FU aqueous solution for the formulations 2 and 3 at the same temperature and dissolved fully under stirring condition.

#### 2.2.2. 3D Printing of the Scaffolds

All tested scaffolds were obtained from Innotere (Radebeul, Germany) and 3D printed using a commercial semi-solid extrusion-based 3D printer with the layer height ~100 microns. 3D expansion was facilitated by alternating orthogonal layers. All scaffolds were cylindrical shaped with diameter 5 mm, height 2 mm, and the strand distance was kept at 0.59 mm. Directly after completing the printing process, the cement setting was performed by storing the scaffolds in water solution for a prolonged period at 50 °C. Infill density of the printed scaffolds was kept at 50%.

#### 2.2.3. Drug Coating for 3D Bio-Scaffolds

3D bio-scaffolds were coated with 5-FU solution by Caleva mini coater/drier 2 (Caleva Process Solutions Ltd., Dorset, UK). Each scaffold was coated with 10 mL drug solutions for 40 min using the formulation composition listed in Table 1. The coating temperature was set as 40 °C, the pumping speed and the agitator frequency were 3.1 rpm and 15.5 Hz respectively. 

#### 2.2.4. Scanning Electron Microscopy (SEM)

The surface and cross-section of the coated and uncoated blank bio-scaffolds were evaluated by using SEM (JEOL Ltd. JSM-820, Tokyo, Japan), which produced a 15-kV acceleration voltage. The entire surface and each region (apical, middle, and coronal) of each canal were examined at magnifications ranging from ×20 to ×1000. The micrographs depicting a magnification of ×200 were chosen for the morphological characterization.

#### 2.2.5. Confocal Microscopy Analysis for 3D Bio-Scaffolds

Confocal microscopy Leica SP8 (Leica Microsystems Ltd., Milton Keynes, UK) was also applied for surface analysis. The wavelength was chosen as 458 nm. Microscope frame is Leica DMi6000 and 20×/0.75 objective was chosen. Live experiment support which heated live chamber (37 °C) and 5% CO_2_ (humidified) was on throughout the analysis. LAS X (Leica Microsystems (UK) Ltd., Milton Keynes, UK) was used as the acquisition software.

#### 2.2.6. Differential Scanning Calorimetry (DSC)

DSC was used to investigate the thermal behavior of coating materials containing 5-FU. The DSC traces of formulations F1 to F3 (the solutions were dried before performing DSC) plus individual materials were conducted using DSC 4000 with aluminum DSC pans (PerkinElmer, Waltham, MA, USA). In order to convert the solutions to powder form to do DSC experiment, coating solutions of formulations F1 to F3 were heated to 80 °C and the temperature was kept for around 20 min (this time was enough to get dry powder). Then, the dried sample was ground into powder using mortar and pestle. A certain amount of formulation powders (5 mg) was placed into the DSC pan and sealed with a lid. Each sample was analyzed from 30 to 400 °C at a scanning rate of 10 °C/min under nitrogen gas. Indium was used to calibrate the DSC for both melting and enthalpy.

#### 2.2.7. X-Ray Powder Diffraction (XRD)

Siemens D500 X-ray Powder Diffraction (XRD) system (KS Analytical Systems, Aubrey, TX, USA) was used to assess the solid-state of the coated scaffolds. XRD test was done at 5–50 theta, the increment was set as 0.1. The data was analyzed by OEM software and reformed by Excel.

#### 2.2.8. FTIR Studies

In order to explore any changes in the molecular level of 5-FU in formulations F to F3, FT-IR equipped with a Universal ATR (Perkin Elmer’s Spectrum One, Waltham, MA, USA) was used. Preceding to analysis, methanol was used to clean the instrument to remove any residual chemicals left on the apparatus, after which a few milligrams of each of the formulations (solutions were dried completely to get powder forms) was used with a pressure of around 70 bar. Each of the samples was scanned three times over a range of 4000 cm^−1^ to 500 cm^−1^.

#### 2.2.9. Texture Analysis

The strength (ultimate compressive strength) of the coated and uncoated scaffolds was tested by texture analyzer (Stable Micro Systems, Surry, UK) with a 25 mm diameter cylinder probe. The test speed was set at 0.03 mm/Sec and the test method was set as “Return to Start” with compression mode. The data was analyzed by Exponent software.

#### 2.2.10. Cancer Cell Culture with 3D Bio-Scaffolds

Scaffolds were transferred to a 12 well plate and UV sterilized. Cells were plated onto the scaffolds at a density of 0.4 × 10^5^ in 10% DMEM and placed into a humidified incubator at 37 °C (5% CO_2_). 24, 96,120 h after plating, the scaffolds were transferred to a new well and washed three times with PBS. Original wells were trysinized and cell count obtained. 0.4 × 10^5^ cells were seeded onto transferred scaffolds. Images were taken prior to trypsinization at 4× magnification using a Floid Cell Imaging Station (ThermoFisher Scientific, Waltham, MA, USA).

#### 2.2.11. Dissolution Studies and Drug Quantification

Dissolution in vitro test was carried out using USP dissolution apparatus II, paddle method (708-DS, Agilent Technologies, Santa Clara, CA, USA) to investigate the drug release pattern from various scaffolds (F1, F2 and F3). The dissolution medium was phosphate buffer (PBS pH 7.2, 900 mL) maintained at 37 °C with a rotation speed of 100 rpm. The coated scaffolds were placed in the dissolution medium and at different time intervals the medium was pumped into UV (Cary 60 UV-Vis, Agilent Technologies, Santa Clara, CA, USA) and read the absorbance of 5-FU at a wavelength of 256 nm. The experiment was carried out in triplicate (*n* = 3) and the release profiles were plotted as a percentage of cumulative drug release versus time.

In order to quantify the amount of drug deposited on each scaffold during the coating process, each scaffold was placed in 50 mL phosphate buffer and sonicated for 20 min. The preliminary results showed that 20 min was enough to dissolve all the drug deposited on scaffolds. The final solution was diluted to be readable in the UV/Vis spectrophotometer at a wavelength of 256 nm. 

## 3. Results and Discussion

### 3.1. 3D Printed Scaffolds and Its Surface Morphology

It has been reported that 3D printing can be an ideal approach to fabricate various micro/macroscale intricate structures owing to its outstanding repeatability and reproducibility which is assisted by a computer-aided method. This emerging technology has started a new era of designing and manufacturing of tissue engineering cell-laden substitutes and biological constructs [25,26]. In this study, CPC scaffolds with interconnected pores (diameter 5 mm, thickness 2 mm) printed using calcium phosphate semi-solid paste (α-tricalcium phosphate and calcium-deficient hydroxyapatite) exhibited rough surface morphology which could be attributed to the microcrystalline morphology of the CPC. An optimized 3D printing process involved utilization of a commercially available semi-solid extrusion-based 3D printer with a maximum print resolution (layer height) of 100 microns. All obtained scaffolds exhibited excellent shape fidelity with interconnected homogenous pores, acceptable process parameters such as syringability, ease of extrusion and bioink malleability.

**Figure 2:**
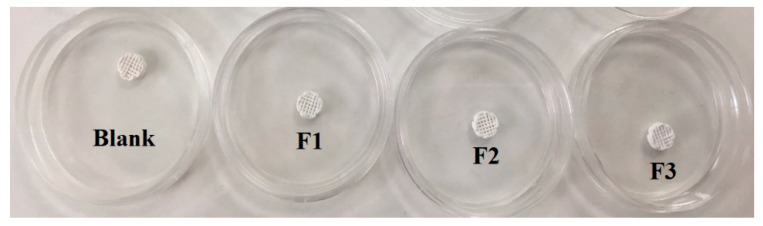
Uncoated/blank and F1–F3 coated scaffolds.

**Figure 3:**
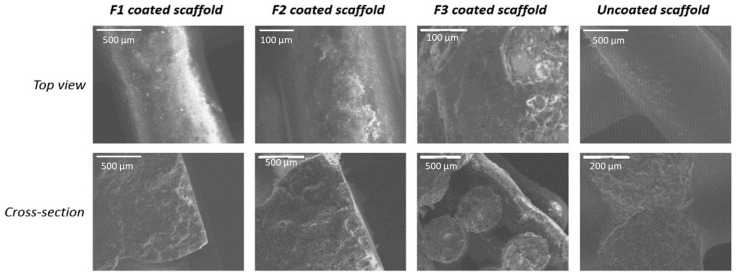
SEM images of coated and uncoated calcium phosphate cement (CPC) scaffolds.

The blank/uncoated CPC scaffold and the other three scaffolds which were coated with formulations F1, F2, and F3 respectively were illustrated in Figure 2. Compared to those coated ones and the blank/uncoated one, these scaffolds show no obvious difference from their physical appearance. The surface morphology of the coated scaffolds (diameter 0.5 cm) via SEM are shown in Figure 3. The top view images (four images above) illustrates that the scaffolds coated with F1 and F2 formulations have a relatively smooth surface. This could be due to the deposition of a homogenous layer of polymeric solution all over the scaffolds. The images of the cross-section were obtained by cutting the CPC scaffold across. Compared to the uncoated CPC scaffold, the presence of a thin polymeric coating layer on each coated scaffold exhibited some adsorbed particles like morphology on the surface which is not visible on the uncoated scaffold.

### 3.2. Confocal Microscopy for 3D Bio-Scaffolds

**Figure 4:**
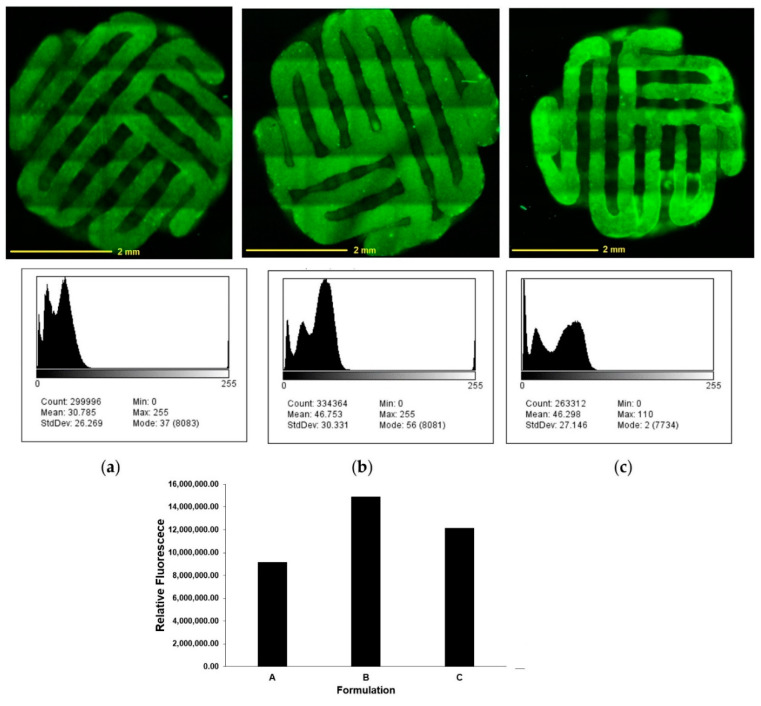
Confocal microscopic images of drug solution coated scaffolds with (**a**) F1, (**b**) F2, and (**c**) F3 (top panel), histograms showing intensity profiles as a function of fluorescence distribution (mid and lower panel).

An advanced surface analysis conducted via confocal microscopy revealed a homogenous distribution of the drug throughout the surface of the scaffolds, represented by the dark green pattern (only the drug is fluorescent in the formulations) (Figure 4). Comparing the three scaffolds coated with different 5-FU formulations showed that F3 coated scaffold (Figure 4c) gives the brightest signal. While the scaffolds coated with F1 and F2 solutions (Figure 4a,b) gives more homogenous results. This is due to the fact that PEG contained in F3 has the highest elongational viscosity which leads to the high viscoelasticity [27,28]. The higher solution viscosity results in the larger thickness deviations. Therefore, a larger amount of the polymer from F3 deposits on the surface of the scaffolds after the coating process. Due to higher viscosity of PEG solution, it led to the higher coating thickness in F3 but with slightly less homogeneity compared to that of scaffolds coated with i.e., F2 solution. Histograms (Figure 4 mid and lower panel) show distribution of fluorescence over the selected area for F1, F2, and F3 (results interpreted by commercial ImageJ). However, the higher standard deviation values are due to the fact that the software also counts backgrounds signal. Nonetheless, this provides sufficient insights to the intensity profile of the homogenous fluorescent 5-FU coating on the surface of all CPC scaffolds.

### 3.3. DSC Analysis for Coating Ingredients

**Figure 5:**
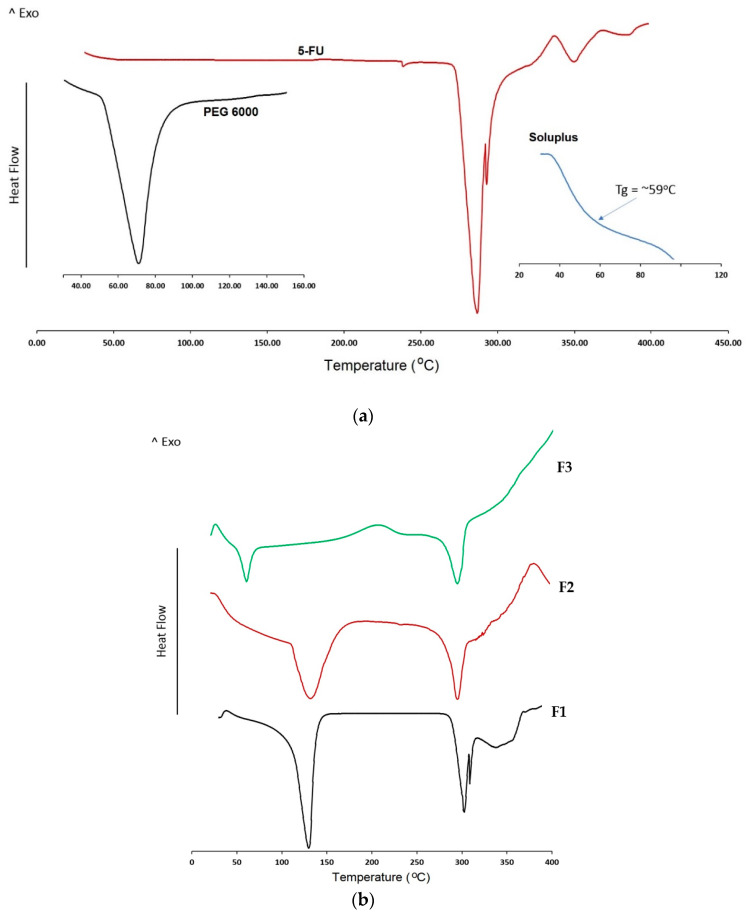
Differential scanning calorimetry (DSC) data of (**a**) bulk drug, Soluplus, and PEG 6000, (**b**) scaffold formulations (F1–F3).

DSC traces for coating ingredients, API and formulations are shown in Figure 5. 5-FU DSC traces show a sharp peak at around 286 °C which corresponds to its melting peak. The second broad peak around 350 °C could be attributed to the thermal degradation of 5-FU. Similarly, PEG 6000 exhibits a sharp endotherm at 68 °C corresponding to its melting point. As it can be seen from DSC traces of all formulations in Figure 5b, it is clear that the melting peak of 5-FU around 280 degrees is present in all the formulations (F1–F3). Comparing the thermal events of the bulk 5-FU and formulation F1 indicates that the peak around slightly higher than 100 °C is due to the evaporation of deionized water left in the coating formulation. The other two formulations (F2 and F3) show a similar thermal event because they were made by a similar evaporation technique from those coating solutions. Formulation F2 containing 5-FU and Soluplus show a combined peak corresponding to the degradation of 5-FU and Soluplus around 350 °C. In addition, data from formulation F3 show a sharp peak around 65 °C, which may be due to the melting of PEG 6000. Therefore, there is no interaction between the components and 5-FU. This indicates that the selected polymers could be suitable polymers to be used along with 5-FU in the scaffold formulation.

### 3.4. XRD Analysis of the Coated Scaffolds

**Figure 6:**
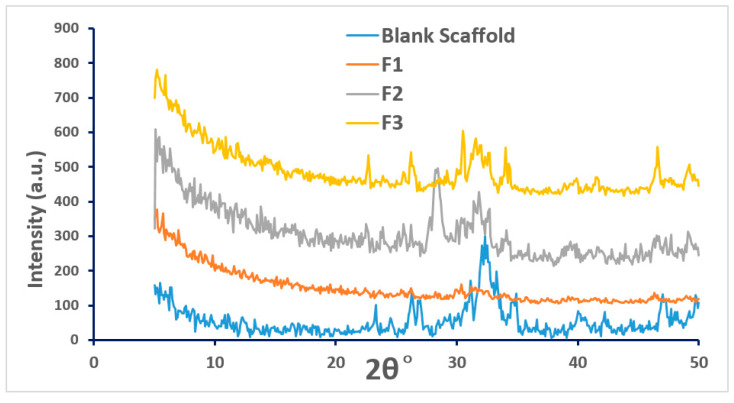
XRD data of blank scaffold (uncoated) and coated scaffolds: F1 (coated with 5-FU), F2 (coated with 5-FU and Soluplus, F3 (coated with 5-FU, Soluplus and PEG 6000).

All CPC scaffolds are synthetic, porous, biocompatible as well as bioresorbable bone substitute materials consisting of α-tricalcium phosphate and microcrystalline hydroxyapatite phases as confirmed via XRD analysis. The XRD data in Figure 6 illustrates that there was no crystal formation on the surface of the CPC scaffolds during the coating process with those three 5-FU drug solutions. The coated scaffolds remain amorphous throughout. The XRD data in Figure 6 illustrates that both blank scaffold and scaffolds coated with solutions F1 to F3 show semi-crystalline structure (or partially amorphous) which is an indication of no major changes in the crystallinity of the scaffold before and after the coating process.

### 3.5. FTIR Analysis

**Figure 7:**
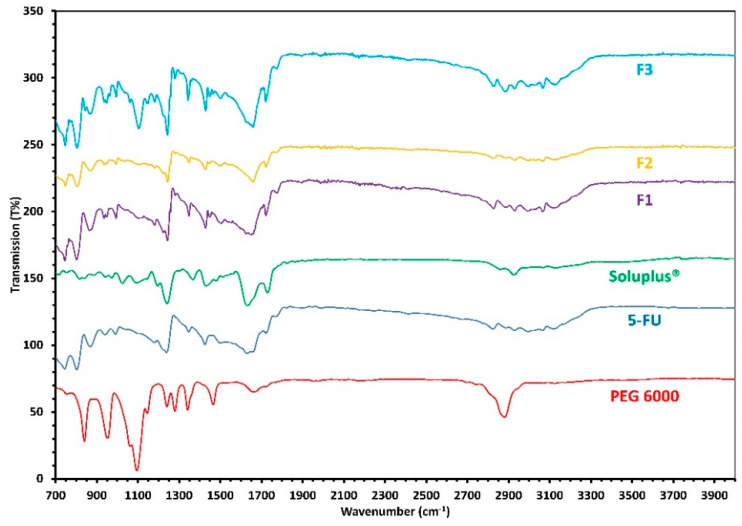
FTIR spectra of bulk materials and formulations F1, F2, and F3.

An FTIR analysis was conducted to analyze any potential interactions between the various components used for coating the CPC scaffolds. It is expected that any potential interaction between the drug and polymer will be reflected by the appearance of any additional bands, alterations in wavenumber position or potential broadening of functional groups when compared with the bulk materials. From the FTIR analysis (Figure 7), it can be seen that there are no major interactions between 5-FU and the polymer solutions used for coating. The absorption band of the bulk compounds are retained in the formulations and helps confirm the absence of chemical interactions between the components. However, a weak intermolecular interaction can be seen between 5-FU and Soluplus^®^ in formulation F2 which is represented by the reduction in the intensity of the 5-FU carbonyl band at 1650 cm^-1^ region. This reduction in the intensity of the band only occurs at higher concentrations of Soluplus^®^ i.e., when the ratio between 5-FU and Soluplus is 1:1 in formulation F2 but cannot be seen when the concentration of Soluplus^®^ is lowered in formulation F3.

This could possibly be due to the presence of both amine and the carboxyl group in the coating formulations. It has been reported that during the FTIR process within carbonyl (COO-) groups two CO bands resonate. As a result, the characteristic CO absorption band can sometimes be replaced by an auto-symmetrical vibration of the COO- group from the polymer [29]. Nonetheless, this suggests that the reduction in the intensity of the 5-FU band at 1650 cm^−1^ in F2 is due to the presence of the excessive amount of Soluplus in the formulation (F2) which may form a weak interaction with the drug but when the amount is lower such as in F3, this interaction disappears.

### 3.6. Texture Analysis

**Figure 8:**
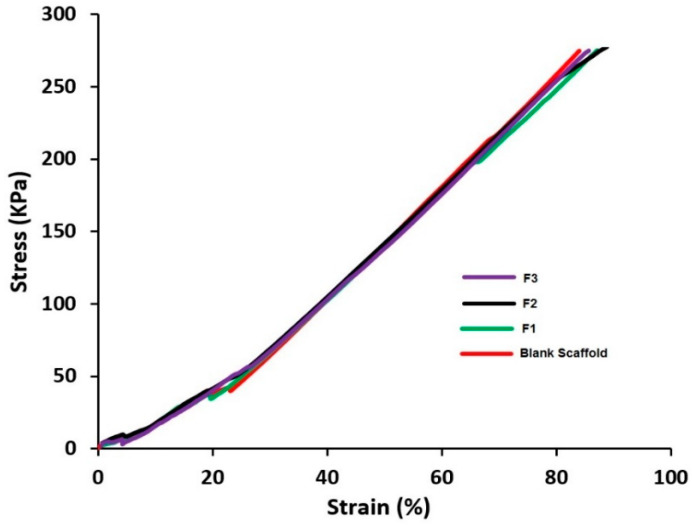
Stress–strain curve of blank scaffold (uncoated) and coated scaffolds: F1 (coated with 5-FU), F2 (coated with 5-FU and Soluplus, F3 (coated with 5-FU, Soluplus and PEG 6000).

Texture analysis was conducted to see the effect of coating on the strength of the scaffolds. According to the data from Figure 8 shows the stress/strain profile of the CPC scaffolds. Mechanical properties such as ultimate tensile strength—the maximum stress that a material can survive under increasing strain before breaking, can be determined from the results presented in Figure 8. The ultimate tensile strength of blank scaffold is 274.43 kPa with a maximum strain of 84%. Interestingly none of the coating formulations have changed the stress–strain profiles of the 3D printed CPC scaffolds significantly. However, a slight increase in the strain value was observed for F2 (85.5%) which could be due to the presence of excessive viscous Soluplus polymer in the coating formulation. In F3 with the decrease of Soluplus content the strain value decreases while the tensile strength is unaffected. In all cases, there is no significant change observed in the mechanical properties of the scaffolds after coating.

### 3.7. In Vitro Dissolution Studies

**Figure 9:**
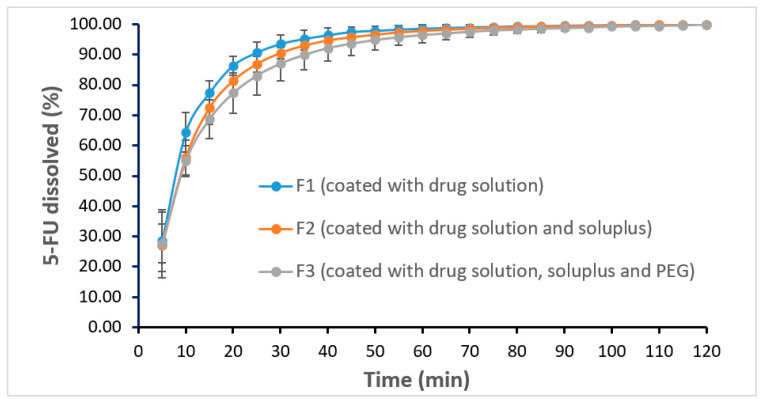
In vitro dissolution profiles of 5-FU from the coated scaffolds with different formulations.

Dissolution profiles of scaffolds coated with various formulations are shown in Figure 9. The results showed that all three coated scaffolds are able to release the entire drug within 2 h. The drug release rate is faster in F1 compared to that of F2 and F3. This was expected as formulation F1 has no polymer, whereas formulations F2 and F3 contained polymers which can potentially act as a barrier to affect the drug release. This slight delay in drug release from F2 and F3 could be attributed to the chemistry of the amphiphilic polymer, Soluplus^®^ which tends to retard the release of the sparingly water-soluble drug i.e., 5-FU upon swelling in the dissolution media [30].

As the polymers used in the coating of the scaffolds are hydrophilic polymers, therefore, these polymers cannot slow down the drug release for a longer time. It is obvious if a longer drug release is needed, it is suggested to use water-insoluble polymers such as ethyl cellulose. Nonetheless, efficient drug release profiles from the coated scaffolds indicates that the deposition of the drug from the coating solutions on each scaffold was achieved successfully.

When the amount of drug deposited on each scaffold was determined, the results showed that the scaffold coated with F1 formulation contained more drug (40.69 ± 1.93 mg) compared to scaffolds coated with formulations F2 (38.49 ± 1.06 mg) and formulations F3 (32.00 ± 2.02 mg), although the initial concentration of drug was kept constant in all coating solutions. This could be due to the change of solid content percentage in the formulations i.e., F1 contains 2.5% *w*/*w* solid whereas both F2 and F3 coating double the amount. Moreover, the viscosity of the coating formulations may play a key role as to the deposition layer thickness, amount of the coating solution and thus the actual amount of drug on the scaffolds. Nevertheless, in all cases, a sufficient amount of 5-FU (>30 mg/scaffold) was successfully deposited.

Drug release kinetics analysis was performed and 4 main kinetics of drug release namely zero-order release, first-order release, Higuchi and Peppas models were considered [31]. The results showed that all formulations followed first order release kinetics with r^2^ values of 0.980, 0.989, and 0.988 for F1, F2, and F3, respectively.

### 3.8. Cell Culture with 3D Bio-Scaffolds

**Figure 10:**
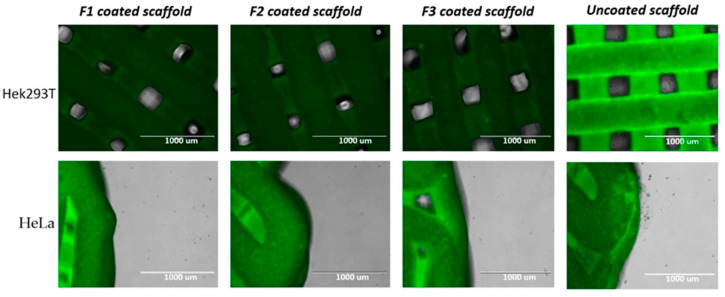
Microscopic images of cell cultured coated and uncoated CPC scaffolds.

**Figure 11:**
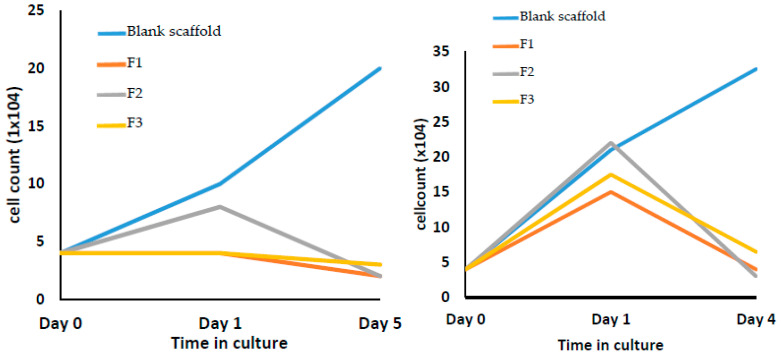
In vitro cell culture assessment of CPC blank scaffold and uncoated scaffold: F1 (coated with 5-FU), F2 (coated with 5-FU and Soluplus), F3 (coated with 5-FU, Soluplus and PEG 6000), with GFP labelled Hek293T (**left**) and HeLa (**right**) cells.

In vitro cell culture studies in two different cell lines (Hek293T and HeLa) were conducted and the results showed a significant reduction of the growth in the number of the cells (Figure 10 and Figure 11). According to the images of the three 5-FU coated scaffolds in Figure 10, there are no visible cells that can be found around the surface of the scaffold. While the blank scaffold (uncoated) was surrounded by living cells. Figure 11 illustrates that the uncoated scaffold did not show any inhibition in the growth of either cell line reflected by an increase in the number of cells by 4–6 fold (after 5 days). In contrast, all formulations showed a significant cell growth inhibition. F2 and F3 coated scaffolds seen to have similar inhibition abilities in terms of reduction in the growth of cells as this is represented by the figures. Although the scaffold coated with F1 shows a relatively weaker effect, the cell counts still went down to near 0 after 4 or 5 days. Nonetheless, it can be claimed that the developed 5-FU coated 3D printed scaffolds can successfully be used as bone graft materials to treat bone cancer and to deliver immediate potential effects for personalized medical solutions.

## 4. Conclusions

CPC scaffolds coated with different 5-FU formulations were created and evaluated in this research. The surface analysis conducted by SEM, confocal microscopy, and XRD illustrate the homogeneous drug-coated outcomes and the amorphous surface of the coated scaffolds. DSC analysis of the coating ingredients shows that there are no chemical reactions occurring between the coating ingredients during bone graft before or after the coating process. Similarly, FT-IR analysis indicated no significant interaction between drug and polymers, though a nominal interaction was observed in F2 due to the higher concentration of Soluplus presents in the formulations which disappeared in F3. Dissolution studies showed that all the coated scaffold released all the drug within 2 h, and the drug release pattern followed first-order release kinetics. In addition, the anti-cancer cell studies confirmed the effective cell killing ability of these 5-FU coated CPC scaffolds. In other words, 5-FU coated 3D printed CPC scaffolds can be successfully used as a novel bone graft material and as a personalized drug delivery system in the treatment of bone cancer.

## Figures and Tables

**Table 1:** Coating formulation compositions of 5-fluorouracil (5-FU).

Formulation Code	5-FU (g)	DI Water (ml)	Soluplus (g)	PEG 6000 (g)
F1	0.5	20	0	0
F2	0.5	20	0.50	0
F3	0.5	20	0.25	0.25

## References

[1] Lepowsky E., Muradoglu M., Tasoglu S. (2018). Towards preserving post-printing cell viability and improving the resolution: Past, present, and future of 3D bioprinting theory. Bioprinting.

[2] Murphy S.V., Atala A. (2014). 3D bioprinting of tissues and organs. Nat. Biotechnol..

[3] Moroni L., Boland T., Burdick J.A., De Maria C., Derby B., Forgacs G., Groll J., Li Q., Malda J., Mironov V.A. (2018). Biofabrication: A Guide to Technology and Terminology. Trends Biotechnol..

[4] Park J.Y., Jang J., Kang H.-W. (2018). 3D Bioprinting and its application to organ-on-a-chip. Microelectron. Eng..

[5] Kang H.-W., Lee S.J., Ko I.K., Kengla C., Yoo J.J., Atala A. (2016). A 3D bioprinting system to produce human-scale tissue constructs with structural integrity. Nat. Biotechnol..

[6] Choi Y.-J., Kim T.G., Jeong J., Yi H.-G., Park J.W., Hwang W., Cho D.-W. (2016). 3D Cell Printing of Functional Skeletal Muscle Constructs Using Skeletal Muscle-Derived Bioink. Adv. Healthc. Mater..

[7] Kim B.S., Lee J.-S., Gao G., Kim Y.K. (2017). Direct 3D cell-printing of human skin with functional transwell system. Biofabrication.

[8] Gudapati H., Dey M., Ozbolat I.T. (2016). A comprehensive review on droplet-based bioprinting: Past, present and future. Biomaterials.

[9] Lepowsky E., Tasoglu S. (2018). 3D Printing for Drug Manufacturing: A Perspective on the Future of Pharmaceuticals. Int. J. Bioprinting.

[10] Kenny S.M., Buggy M. (2003). Bone cements and fillers: A review. J. Mater. Sci. Mater. Med..

[11] Xu H.H., Wang P., Wang L., Bao C., Chen Q., Weir M.D., Chow L.C., Zhao L., Zhou X., Reynolds M.A. (2017). Calcium phosphate cements for bone engineering and their biological properties. Bone Res..

[12] Kilian D., Ahlfeld T., Akkineni A.R., Bernhardt A., Gelinsky M., Lode A. (2020). 3D Bioprinting of osteochondral tissue substitutes – in vitro-chondrogenesis in multi-layered mineralized constructs. Sci. Rep..

[13] Ahlfeld T., Cubo-Mateo N., Cometta S., Guduric V., Vater C., Bernhardt A., Akkineni A.R., Lode A., Gelinsky M. (2020). A Novel Plasma-Based Bioink Stimulates Cell Proliferation and Differentiation in Bioprinted, Mineralized Constructs. ACS Appl. Mater. Interfaces.

[14] Trombetta R.P., Ninomiya M.J., El-Atawneh I.M., Knapp E.K., Bentley K.L.D.M., Dunman P.M., Schwarz E.M., Kates S.L., Awad H. (2019). Calcium Phosphate Spacers for the Local Delivery of Sitafloxacin and Rifampin to Treat Orthopedic Infections: Efficacy and Proof of Concept in a Mouse Model of Single-Stage Revision of Device-Associated Osteomyelitis. Pharmaceutics.

[15] Diasio R.B., Harris B.E. (1989). Clinical Pharmacology of 5-Fluorouracil. Clin. Pharmacokinet..

[16] CAMEO Chemical Datasheet, Flurouracil. https://cameochemicals.noaa.gov/chemical/5005.

[17] (2010). BASF, The Chemical Company, Soluplus, Technical Information. http://www.rumapel.com.ar/pharma_excipientes/ficha_tecnica/Soluplus.pdf.

[18] BASF SE 2020 Soluplus—For Better Solubility and Bioavailability. https://pharmaceutical.basf.com/en/Drug-Formulation/Soluplus.html.

[19] Kanmani P., Lim S.T. (2013). Synthesis and characterization of pullulan-mediated silver nanoparticles and its antimicrobial activities. Carbohydr. Polym..

[20] Maniruzzaman M. (2019). 3D and 4D Printing in Biomedical Applications: Process Engineering and Additive Manufacturing.

[21] Malda J., Visser J., Melchels F.P., Jüngst T., Hennink W.E., Dhert W.J.A., Groll J., Hutmacher D.W. (2013). 25th Anniversary Article: Engineering Hydrogels for Biofabrication. Adv. Mater..

[22] Sakai S., Yoshii A., Sakurai S., Horii K., Nagasuna O. (2020). Silk fibroin nanofibers: A promising ink additive for extrusion 3D bioprinting. Mater. Today Bio.

[23] Lee J., Hong J., Kim W., Kim G.H. (2020). Bone-derived dECM/alginate bioink for fabricating a 3D cell-laden mesh structure for bone tissue engineering. Carbohydr. Polym..

[24] Wu Y., Heikal L., Ferns G., Ghezzi P., Nokhodchi A., Maniruzzaman M. (2019). 3D Bioprinting of Novel Biocompatible Scaffolds for Endothelial Cell Repair. Polymers.

[25] Pina S., Ribeiro V.P., Marques C.F., Maia F.R., Silva T.H., Reis R.L., Oliveira J. (2019). Scaffolding Strategies for Tissue Engineering and Regenerative Medicine Applications. Materials.

[26] Van Belleghem S., Torres L., Santoro M., Mahadik B., Wolfand A., Kofinas P., Fisher J. (2020). Hybrid 3D Printing of Synthetic and Cell-Laden Bioinks for Shape Retaining Soft Tissue Grafts. Adv. Funct. Mater..

[27] Kistler S.F., Schweizer P.M. (1997). Liquid Film Coating: Scientific Principles and Their Technological Implications.

[28] Hong Y. (2016). Electrospun Fibrous Polyurethane Scaffolds in Tissue Engineering. Advances in Polyurethane Biomaterials.

[29] Maniruzzaman M., Douroumis D. (2014). An in-vitro-in-vivo taste assessment of bitter drug: Comparative electronic tongues study. J. Pharm. Pharmacol..

[30] Shi K., Tan D.K., Nokhodchi A., Maniruzzaman M. (2019). Drop-On-Powder 3D Printing of Tablets with an Anti-Cancer Drug, 5-Fluorouracil. Pharmaceutics.

[31] Akbari J., Adrangui M., Farid D., Siahi-Shadbad M.R., Saeedi M., Nokhodchi A. (2000). The effects of various factors on the release rate of a poorly solubledrug (carbamazepine) from hydroxypropyl methylcellulose matrices. S.T.P. J. Pharma. Sci..

